# Beyond Tumor Suppression: Senescence in Cancer Stemness and Tumor Dormancy

**DOI:** 10.3390/cells9020346

**Published:** 2020-02-03

**Authors:** Francisco Triana-Martínez, María Isabel Loza, Eduardo Domínguez

**Affiliations:** Biofarma Research Group, Center for Research in molecular Medicine and Chronic Diseases (CIMUS), Universidad de Santiago de Compostela, Avenida de Barcelona s/n, 15782 Santiago de Compostela, Spain; box_trianas7@hotmail.com (F.T.-M.); mabel.loza@usc.es (M.I.L.)

**Keywords:** cellular senescence, stemness, dormancy, quiescence, senolytic

## Abstract

Here, we provide an overview of the importance of cellular fate in cancer as a group of diseases of abnormal cell growth. Tumor development and progression is a highly dynamic process, with several phases of evolution. The existing evidence about the origin and consequences of cancer cell fate specification (e.g., proliferation, senescence, stemness, dormancy, quiescence, and cell cycle re-entry) in the context of tumor formation and metastasis is discussed. The interplay between these dynamic tumor cell phenotypes, the microenvironment, and the immune system is also reviewed in relation to cancer. We focus on the role of senescence during cancer progression, with a special emphasis on its relationship with stemness and dormancy. Selective interventions on senescence and dormancy cell fates, including the specific targeting of cancer cell populations to prevent detrimental effects in aging and disease, are also reviewed. A new conceptual framework about the impact of synthetic lethal strategies by using senogenics and then senolytics is given, with the promise of future directions on innovative anticancer therapies.

## 1. Introduction

Natural tumor evolution is a complex process, composed of multiple steps (cell-intrinsic tumorigenesis, tumor growth, invasion, and metastasis), cellular phenotypes, microenvironmental treats, and immune system interplay. Pharmacological treatment just adds more complexity to this evolution by the appearance, selection, and exacerbation of specific phenotypes, including senescent tumor cells, quiescent tumor cells, and cancer stem cells. Among these, a new cellular outcome named dormancy has been proposed. Cells in dormancy may promote a more lethal profile relapse of tumor growth, even after many silent years or decades. There is now a large body of clinical and experimental evidence to accept the existence of tumor cell dormancy; however, there are still a number of questions to be addressed about the nature of this kind of cell, including its origin, evolution, and nature. One of the aims of this review is to attempt to understand the nature of dormant tumor cells through the knowledge that we currently have about other tumor cell phenotypes; in particular, from the state-of-the-art on cancer stem cells, because these two phenotypes share some similar characteristics, and on senescence, because senescence is a primary response to pharmacological treatment in cancer (despite apoptosis) and it strongly influences the regulation of stem-like phenotypes.

Since their discovery, cancer stem cells (CSC) have gained a lot of attention, and extensive research has been focused on CSCs since they are not only highly resistant to conventional chemotherapy, but also possess the capacity to regrow a complete tumor after clinical intervention. This last capacity is due to their intrinsic self-renewal capacity. CSCs exist in a most undifferentiated state within tumors; however, there is no consensus about the origin of CSCs. It is proposed that they arise from normal adult stem cells, obtaining the capacity to grow as a tumor by a mutation on specific genes (reviewed in [1]).

The rapid advances in cellular senescence—a highly relevant phenotype in physiology and disease widely involved in eukaryotic organism physiology—make it difficult to keep up with and integrate many of the key concepts and developments. Depending on the biological context, senescence can be a beneficial or deleterious cellular outcome. Senescence is a natural intrinsic response of cells against stress situations, and its activation avoids the proliferation of potentially malignant cells in an irreversible fashion, so it has been considered a primary tumor suppressor mechanism [2]. Senescence is also associated with the resolution of fibrosis in a mechanism that includes senescent cell recognition by the immune system [3]. In addition, embryonic developmental senescence has been observed to participate in tissue remodeling and the formation of macro structures like limbs or mesonephros (reviewed in [4]). On the other hand, senescence accumulation in tissues promotes a state of chronic inflammation linked with a reduced physiological fitness during aging (reviewed in [5]). This inflammatory microenvironment, in combination with the growth factors produced by senescent cells, may promote the proliferation of non-senescent tumor cells or the acquisition of the most aggressive phenotypes like cancer stemness (reviewed in [6]), or, as we propose, cells with the ability to produce tumor regrowth in cancer patients after years of disease-free survival.

Another non-proliferative but harmful phenotype is quiescence. However, as opposed to senescence, quiescence is characterized by reversible cell cycle arrest, promoting, among other characteristics, a high resistance to toxic stimuli, including cancer therapies [7]. In a tumor context, it has been proposed that this state is the prevalent state in the CSC phenotype and putatively on dormant cells. With respect to this view, it has been proposed that dormant cells are a special case of stem cells in a quiescence state. However, based on the cancer evolution fundament, we propose that senescence could act as a source of dormant tumor cells. Therefore, the general aim of this work is to provide a comprehensive perspective on the definition of the fate of tumor cells (senescent or not) and to highlight the translational potential of therapeutic avenues, primarily based on manipulating cellular senescence.

## 2. Cancer Stem Cells

Stem cells possess a self-renewal capacity, give rise to progeny capable of differentiating into other cell types [8,9,10], and hold a high cell plasticity emerging from specific pluripotency genetic programs [11,12,13]. Small populations of cells with active pluripotency programs and a high plasticity, known as cancer stem cells (CSCs), exist in tumors [14,15,16,17]. CSCs were characterized for the first time in acute myeloid leukemia (AML), in which cells with the CD34+/CD38− phenotype exhibited proliferative and self-renewal capacities similar to those of primary stem cells [18]. It is now widely accepted that all tumors have CSCs, although it is still unclear whether these cells arise from primary development defects and are tumor initializers [19,20], or if they evolve from a somatic tumor cell that acquires stemness characteristics [21]. For example, a CSC-like phenotype can be acquired by epithelial-mesenchymal transition (EMT) programs [14,22] or by escaping from senescence [23]. Pluripotency and cellular plasticity promote new physiological traits conferring drug resistance [24,25] and/or angiogenic, invasive, and metastatic potentials [26,27,28].

## 3. Senescence and Stemness Are Relevant in Cancer Evolution

### 3.1. The Senescent Phenotype 

Cellular senescence is a non-proliferative steady state that can be acquired by both primary [2,29,30,31] and malignant cells [32,33,34]. The susceptibility of primary stem cells to enter into a state of senescence was recently described in muscle cells, pituitary tumor cells, and mesenchymal stem cells [35,36,37]. Senescence cell cycle arrest is mainly (but not only) promoted by a sustained DNA damage response (DDR); by oncogenic activation; and by other stress conditions that dramatically change the morphology, gene expression, secretory program [2,34,38,39,40,41]. Senescent cells exhibit a complex secretome (in many cases, a denominated Senescence Associated Secretory Phenotype (SASP)) composed of growth factors, cytokines, chemokines, bioactive lipids, and extracellular matrix remodelers that plays a critical role in the cell cycle, immune response, and tissue remodeling, depending on the physiopathological context [42,43,44].

### 3.2. Senescence Promotes Intrinsic and Paracrine Stemness 

Senescence promotes stem-like reprogramming (a set of pluripotency and stem cell features) in an autocrine and paracrine way. In the last few years, the relationship between senescence and stemness has been intensively explored, including the critical regulation of specific functions of stem cells that drives them into replicative senescence in multiple tissues, including those of the hematopoietic system, intestine, muscle, brain, skin, and germline. Stemness is regulated by a number of signals and genes that influence tumor development (Table 1). It is interesting to note that senescence was described as a physiological mechanism in embryonic development [45,46] and as a mechanism of tissue regeneration [47,48,49]. Likewise, senescent cells from Oncogene-Induced Senescence (OIS) express stem cell-related genes. However, due to their cell cycle arrest, they do not develop clonogenicity or a self-renewal capacity. The SASP, or senescent secretome, promotes an environment in which neighboring cells are favored to express stemness markers [49]. In the same way, SASP can enhance the genetic induction of pluripotency in induced pluripotent stem cell (iPSC) generation in vivo with reprogramming factors [50,51]. Experimental evidence suggests that the accumulation of senescent cells promotes paracrine tumorigenesis through their secretome and favors the appearance of more aggressive stem-like phenotypes [52]. Conditioned media from senescent cells promotes clonogenicity and cancer stemness [53] and the development of subpopulations of cells resistant to chemotherapy [52,54]. As previously mentioned, tumor cells that escape from senescence also acquire preeminent tumorigenicity, drug resistance, and other characteristics of CSCs in a process called senescence-associated stemness (SAS), promoted by the loss of tumor suppressors and the activation of conserved embryonic development pathways as Wnt signaling [23] (see Table 1 for genetic pathways associated with stemness).

### 3.3. Senescence Induction on Stem Cells and Cancer Stem Cells

**Figure 1:**
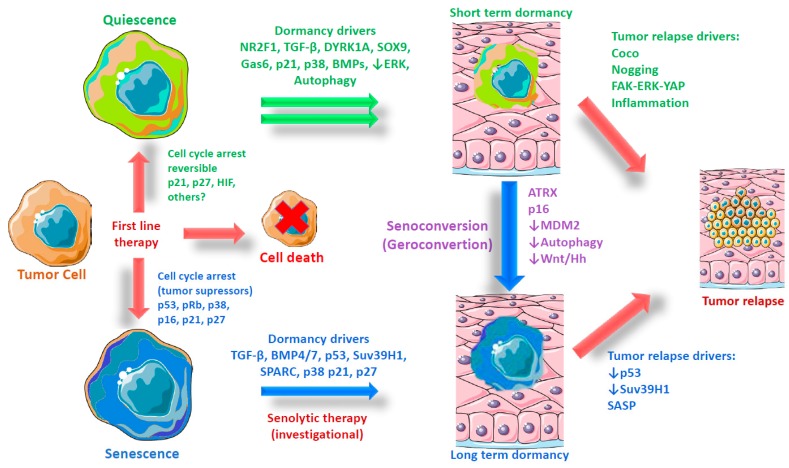
Origin and evolution of dormant tumor cells. Graphical representation of the evolution of tumor dormancy according to two models—quiescence and senescence—including possible triggers and driver genes. Quiescence is the most accepted model for tumor cell dormancy. This model is based on the observation of some molecular triggers in the microenvironment and final effectors that regulate cell cycling. Dormant cells have been observed to express markers such as a low ERK/ high p38 ratio during the activation of other genes like NR2F1 and DYRK1A. However, in some models of dormancy, senescence markers have been noted. This observation, plus the activation genes during senescence shared with quiescence (i.e., TGF-β, BMPs, p21, and p27), leads us to propose an alternative model of tumor dormancy in which senescence is a major phenotype acquired during long-term dormancy. If this assumption is true, it opens the possibility of the use of senolytics to avoid metastatic recurrence.

Previously, it was believed that only proliferative cells were able to enter in senescence, which was considered an intrinsic barrier preventing reprogramming to a more dynamic state [55,56]; however, it is now well-recognized that primary (muscle) stem cells (low proliferative capacity cells) can shift from quiescence to senescence during aging in a process known as geroconversion (henceforth referred to as senoconversion), which is dependent on the overexpression of the tumor suppressor cyclin-dependent kinase Inhibitor 2A Cdkn2a (p16), a well-characterized senescence biomarker that acts by slowing the progression of the cell cycle from the G1 phase to the S phase [35] (see Figure 1). Likewise, the failure of autophagy caused by aging or genetic mutations in cells from young donors also induces senescence in muscle stem cells [57]. Other putative mechanisms inducing senescence in primary and cancer stem cells include DNA damage by chemotherapeutics [37], hypoxia [58], oncogene activation (oncogenic β-catenin) [36], and the dysregulation of microenvironmental growth factors such as TGF-β (transforming growth factor beta) or BMP7 (bone morphogenetic protein 7) [59,60]. In addition to SASP, other factors might be required to induce senescence. The Notch signaling pathway—a highly conserved system mediated through cell-to-cell contact (juxtacrine)—not only has a different gene transcription signature than primary OIS, but is also an essential driver of secondary senescence, a distinct molecular endpoint from OIS [61]. In summary, tumor suppressor and oncogenic pathways regulate senescence and stemness interplay, influencing the microenvironment and transforming cancer cells in heterogeneous populations, according to their cell cycle state.

## 4. Role of Tumor Suppressor and Oncogenic Pathways in Senescence

Since senescent cells are non-proliferative, senescence has been considered an intrinsic mechanism of tumor suppression primarily characterized by the activation of “tumor suppressor pathways” p53–p21 or p38–p16–pRb [2,62,63] (Figure 1). Senescent cells are present in premalignant lesions or primary stages of tumorigenesis; however, senescent cells disappear in the late stage of tumor development [31,33,63]. For these reasons, senescence is thought to be essential for complete oncogenic transformation [63,64]. Likewise, the loss of some oncogenes in developed tumors promotes senescence and rapid tumor size regression, while the specific abrogation of some senescence pathways fails to avoid tumoral regrowth [65,66]. For example, in primary mouse embryo astrocytes, Ras activation is not linked to senescence, supporting the possibility that oncogene activation may not be a widespread mechanism [67]. The modulation of SASP in prostate tumors is a promising strategy to elicit tumor suppression. In vivo, Phosphatase and tensin homolog (PTEN)-null senescent cells secrete cytokines through the Jak2/Stat3 pathway; promote the infiltration of immunostimulatory CD8+ T cells, NK cells, and B cells; and decrease the tumor size and invasion capacity [68]. The pharmacological inhibition of Jak2/Stat3 leads to an antitumor immune response that enhances the efficacy of chemotherapy, suggesting that the oncogene PTEN is involved in immunomodulation of the tumor microenvironment through SASP.

## 5. Therapy-Induced Senescence

Despite the primary induction of apoptosis during cancer treatment and depending on the concentration and time of exposure to pharmacological and physical agents, a senescence phenotype can arise, affecting a large number of cells within the tumor. Treatment with conventional physics methods (i.e., ionizing radiation), conventional chemotherapy that generally produces a DNA damage response, and some target therapies trigger therapy-induced senescence (TIS) [34,69].

TIS is an important outcome since it produces the existence of tumors lacking p53 and pRB; however, senescence induction pathways are redundant in many locations downstream, especially on the final regulators of the cell cycle (i.e., p16, p21, and p27). Due to this, all cancer cells retain the ability to enter on senescence with appropriated stimuli (reviewed in [70]).

Despite the growth suppression of senescence induction, TIS may promote inflammation and angiogenesis and is the cause of some deleterious effects of chemotherapy [71] or even recurrence [6,72,73,74]. Nevertheless, the use of senolytics—molecules that kill specifically senescent cells—has been proven to be effective for avoiding such deleterious effects [71]. In addition, the secretome from primary senescent cells (surrounded or not surrounded by tumor tissue) has major pro-tumorigenic effects [53,75,76], particularly in mesenchymal and stromal cells [77,78,79]; at present, it is not completely clear if these surrounding cells become senescent in response to cancer treatment or other physiological stimuli. Senescence plays a dual role in oncogenesis and tumor suppression, both protective and detrimental, affecting the microenvironment and tissue functionality, and eventually leading to pro-carcinogenic signals. It is necessary to find new senogenic compounds to promote specific senescence on cancer cells and not in primary cells, in order to reduce the negative effects of TIS, and to combine this with senolytics. In this way, a recent report on an experimental treatment for liver cancer cell lines derived xenografts, and described the use of pharmacological screenings to find a novel and specific senogenic and senolytic to promote strong tumor regression [80].

## 6. Senescence Implications in Metastasis

It is well-established that senescence promotes the appearance of tumor cells with a high stemness and promotes EMT [42,81]. In addition, the senescent secretome from tumor and primary cells (especially from epithelial and stromal cells) promotes the optimal microenvironment for the invasion of adjacent tissues and migration to distant sites. Among the factors secreted by senescent cells, the extracellular matrix remodelers [42,82,83] and angiogenic factors, especially the vascular endothelial growth factor (VEGF) [76,84,85], are the most important for the metastasis process. Because of their large and flattened morphology and due to the lack of a proliferation capacity, it is believed that senescent cells remain motionless and therefore do not display invasive behavior. However, senescent tumor cells are frequently present in the front region of the collective invasion of papillary thyroid carcinoma in patients, as well in lymphatic channels and metastatic foci of lymph nodes [86]. In melanoma cells, exposure to the B-raf inhibitor promotes a senescent-like state (including a high p21 expression, senescence-associated heterochromatic foci (SAHF), Promyelocytic Leukemia (PML) bodies activation, and increased activity of SA-β gal); even so, a subpopulation with a high Wnt5A expression was able to colonize the lungs in in vivo tail-vein colony-forming assays [87], indicating that senescent cells can conserve the malignant capacity to migrate to distant tissues.

Additionally, senescent cells can act as chemo-attractants and lead to proliferative tumor cells through factors included in the senescent secretome [86,88]. Moreover, the increase in senescence-associated aging promotes the emergence of pro-metastatic niches in bone, possibly by the secretion of IL-6. Using antibodies against IL-6 was necessary and sufficient to stop senescence-induced osteoclastogenesis and bone metastasis [89]. These findings suggest that senescent cells are involved in invasion and metastasis and predict that interventions targeting cellular senescence and SASP may improve cancer outcomes and reduce the metastatic process (Table 1).

## 7. Tumor Cell Dormancy

One of the major challenges in current cancer treatments is cancer relapse and metastatic recurrences. Metastatic recurrences are the resurgence of tumors in different tissues from which primary cancer arose, months, years, or even decades after the eradication of the primary tumor. Metastatic recurrence is indeed the major cause of death in cancer patients [90]. Like all the characteristics of tumor cells used in their favor, the capacity to spend long periods in diapause is a conserved physiological process, and is especially observed in memory T lymphocytes [91,92]. To survive after chemotherapeutic treatments, dormant tumor cells (DoTC) need to possess a different biology from the normal cells and acquire at least the following characteristics: stress and drug resistance, invasion, a dissemination capacity, and the capacity to enter and exit from diapause. Three models of dormancy have been proposed: (a) cellular dormancy, in which a single cell survives in quiescence; (b) angiogenic dormancy, in which cells remain isolated in environments with a low concentration of oxygen and nutrients; and (c) immunosurveillance dormancy, in which the immune system prevents tumoral re-growth [93].

### Dormancy and Metastatic Recurrence

Dormancy and metastasis are two different concepts, but they are related. Molecular characterization and relevance in disease of the DoTC are currently being extensively reviewed. Both DoTC and metastatic cells present a similar organotropism, but metastatic cells (in their extensive definition) activate proliferation programs in less time [94,95], while DoTC proliferation will take a longer time and may possibly be more sensitive to microenvironmental changes, as determined by its own physiology [96,97,98] (Table 1, oncogenes as the origin of proliferative fate). DoTC are, in fact, a subpopulation of circulating tumor cells (CTC) [99] and a fraction of disseminated tumor cells (DTC) engaged in metastatic niches [100]. The origin of metastatic cells has been associated with the late-stage of tumor development, in which new characteristics are acquired by de novo genetic mutations or selected by chemotherapeutic treatments [94]. However, the best explanation of clinical and preclinical evidence is that metastatic cells are produced and disseminated early on in cancer development [101,102,103]. This situation seems to be no different from DoTC, which develop into lesions prior to the appearance of the primary tumor [92,104,105,106,107]. In this context, it has been demonstrated that dormant cells arise from the primary tumor in specific hypoxic niches [20] by the activation of genes like Nr2f1 and promote heterogenicity (Figure 1). This notion about the existence of cells with long and short diapause is supported by data on D2A1 and D2.0R tumor cell lines derived from murine mammary hyperplastic alveolar nodules. Both cell lines produce lung metastasis, although the D2A1 cell line evolves in a short period of time (1-3 weeks), while D2.0R takes a long time to evolve (4 months), after recipient injection [98,108,109,110]. Dormant pancreatic tumor cells in which oncogenic drivers such as KRAS and c-myc are mutated display increased autocrine IGF1/AKT signaling that controls survival and the pharmacological inhibition of IGF-1R reduces the residual disease burden and cancer recurrence [111]. For reviews on the immune targeting of dormancy and metastasis, see references [97,112].

## 8. Molecular Mechanisms Underlying Dormancy, Quiescence, and Senescence

Which is the genetic identity of these cellular states? One of the first attempts to reveal DoTC identity compared them with cancer stem cells (CSCs) [113]. As already mentioned above, CSCs [114] and DoTC [90] share similar characteristics (e.g., a resistance to chemotherapy drugs, high migration capacity, and tumor initiation and regrowth ability). CSCs are in a quiescence (or G0)-like state [115] and can restart proliferation with a better disposition and in less stringent conditions than those required by DoTC [116,117,118]. In fact, DoTC expresses some stemness regulation genes related to adult primary stem cells, such as TGF-β and BMP [119,120,121]. Likewise, the dissemination of early breast tumor cells with a DoTC phenotype requires the activation of cellular plasticity pathways (e.g., Wnt and RANK) to promote migration, but not proliferation [107,122]. The pluripotency program is also active in dormancy neck squamous cell carcinoma, in which SOX2, SOX9, OCT4, and NANOG genes are upregulated [123] (see Table 1 for a summary of the genetic origin and relevant trigger signals for proliferation and dormancy fates). Nevertheless, DoTC do not accomplish a complete EMT [124], and upon specific environmental conditions, can restart proliferation [125].

### Primary Signaling in Dormancy and its Relevance for Cancer Stem-Like and Senescent Cells

There is a consensus about the existence of dormancy pathways resembling quiescence [90]. Evidence suggests that DoTC have the constitutively active “stress pathway” CDK4/6–p38α/β and inactive “proliferation” pathway Ras–MEK–ERK1/2 [126,127]; whilst other stress pathways can be active during dormancy, like ATFα6-mTor [128]. During adult pluripotency, stem cells activate the Ras–ERK pathway to escape from quiescence and proliferate, in turn suppressing the pluripotency program [129,130], while CSCs can retain some of the stem characteristics after Ras-ERK activation [131,132,133], fitting well with the DoTC profile (Figure 1 shows the involvement of ERK as a driver for quiescence-dormancy conversion and in dormancy-tumor relapse). If pluripotency is genetically induced with Yamanaka’s factors (i.e., Myc Oct4, Sox2, and Klf4), the Ras pathway plays a dual role; in a malignant context, Ras inhibits pluripotency acquisition, while in an oncogenic context, it promotes it [134]. On the other hand, the activation of p38 cellular stress reduces pluripotency and self-renewal in CSCs [135,136,137,138]. Are DoTC and CSCs identical in essence? Both proliferation and stress pathways (Ras–ERK and MKK–p38) have an important role in senescence. As mentioned above, oncogene activation produces senescent cells (OIS) in premalignant lesions, with the Ras mutation being the most common [31,33]. However, once senescence is established, Ras expression does not seem to have direct consequences on the senescent phenotype. Furthermore, its prolonged activation produces negative feedback that reduces the stimulation of the RAS–MEK–ERK pathway [139]. On the other hand, during physiochemical stress situations, primary cells go to senescence by the activation of p38α–p16–pRb [140,141]. We reason that even though DoTC conserve some stem features, they cannot be considered as CSCs in a typical way. The evidence suggests the possible existence of CSC subtypes and perhaps that is also true for DoTC. Puig and cols. [142], used an elegant experimental design to tag colorectal cancer cells (CRC) with the doxycycline-inducible histone H2Be–GFP. After a pulse of doxycycline and some cycles of cellular division, those cells retaining the fluorescence reporter were isolated. The gene expression profile of these slow-cycling cancer cells (SCCCs) was enriched for drug detoxification, stemness, hypoxia, or crosstalk, with the immune system denoting it’s stem cell nature, which might be a starting point for characterizing the underlying molecular signature of putative dormant cells. However, isolated SCCCs from spheroids have the same proliferative behavior in vitro (from single-cell samples) and in vivo than their counterparts (rapid RCCCs), suggesting the different nature of these cells and the relevance of the microenvironment for establishing the signaling of dormancy.

## 9. Dormant and Senescent Cells: One and the Same or Another Kind?

The elevated activation of p38 in DoTC should make them lose their stem features; however, it is partially conserved. This opens the opportunity to ask relevant questions: Can the DoTC be in a different state to quiescence? Which other diapause state is compatible with a high level of p38 activation and retention of stemness? We must keep in mind that when a cell enters in senescence, it seems to acquire some stem features [23,49], so, it is justified to ask if DoTC are in a senescence-like state or if senescence in tumor cells should be considered a different kind of dormancy. Reports from Kobayashi and cols. [60], Sharma and cols. [143], and Bartosh and cols. [144] (revised further) suggest that, in fact, the state of dormancy is acquired through some kind of senescence. This observation was made using two different models induced by two different mechanisms (including signaling from BMPs and entosis, which is the process whereby cells are internalized into neighboring cells, forming ‘cell-in-cell’ structures), with both occurring putatively in the same site (bone marrow). A study from Kobayashi and cols. [60] also suggests the possibility that they can be in a state of stem cells (quiescence) and senescent at the same time, since DoTC are selected using stem cell markers after exposing them to BMP.

### 9.1. TGF-β Family Factors in Dormancy and Senescence

A very interesting factor with proven activity during cancer cell dormancy is TGF-β, which is a multifunctional regulatory cytokine. Senescence induction mediated by TGF-β is widely documented in tumor and primary cells [145,146,147,148,149]. High TGF-β2 signaling, as found in bone marrow, promotes the activation of p38α/β stress and a dormancy state in tumor cells by the activation of TGF-βRI yRII receptors, and through the phosphorylation of p27 [120,150]. Similarly, Bone Morphogenic Protein BMP4/7, another TGF-β family member secreted by bone stromal cells, participates in dormancy induction in prostate and triple-negative breast cancers through the activation of p38, p21, p27, and NDRG1 [60,119,151]. Some reports also suggest that TGF-β and BMP induce quiescence in prostate cancer [152] and other solid tumors [153], as well as in primary cells [154]. However, breast and prostate tumor cells can be induced into dormancy by BMP7 secreted from bone stromal cells through BMPRII (BMP receptor II), using β-gal staining as positive markers of senescence and genetic in vivo models of suppression of BMP receptor signaling [60,155]. Prostate tumor cells in senescence show p38 phosphorylation activity and low ERK activity (even in the presence of an epidermal growth factor (EGF), a very well-known ERK activator), proposed as a marker of cells undergoing dormancy. This dormant-senescent equilibrium state is directly related to the activity of N-myc downstream-regulated gene 1 (NDRG1), which may open up new opportunities for targeting as a regulatory mechanism. Interestingly, blocking BMP7 signaling in vitro and in vivo reverses senescence and causes cells to restart proliferation [60]. NDRG1 and BMP4 also induce senescence in other tumor and primary cells [156,157,158,159]. Finally, TGF-β and other members of its family (like BMP6, BMP2, Inhibin A, and GDF15) are fundamental factors present in the senescent secretome [160] essential for the paracrine induction of senescence [43,161,162] and recognized as dormancy drivers (Figure 1). It is also interesting that, during aging, TGF-β-mediated signaling induces senescence in bone-derived mesenchymal stromal/stem cells through p21 activation [59,163].

### 9.2. CDK Inhibitors in Dormancy, Quiescence, and Senescence

Cell cycle arrest is carried out by CDK inhibitors (CDKi), especially p21 and p27 producing quiescence upon TGF-β-induced dormancy [60,120,164]. However, these signaling pathways controlling the cell cycle during quiescence are overlapped with the senescence induction process [165,166], especially in tumor cells lacking active p16 [167], in which senescence primarily depends on p21 and p27. As mentioned above, there is still controversy on the role of CDK4/6 inhibitor drugs (e.g., Palbociclib or Abemaciclib) in senescence or quiescence induction [168,169,170]. Senescence induction depends not only on the chronicity of treatment, but also on other factors, such as proteasome inactivation [171] and autophagy [172]. DNA-damaging chemotherapeutics induce senescence mainly activated by the p53-p21 pathway, although, if p53 is deactivated, cells can escape from senescence, resuming proliferation and not entering in quiescence (reviewed in [173]). On the other hand, p27 participates in senescence induction, but more importantly, provides the ability to avoid cells to escape from senescence by blocking CDK1, an effect not observed for p21 [174]. These mechanisms might be clinically relevant, since high levels of p27 are correlated with a better prognosis in patients with different types of tumors [175], including prostate cancer [176], which is associated with the longest dormancy periods.

### 9.3. Emerging Molecular Mechanisms Controlling Quiescent or Senescent Fate

If we consider as a premise that not only are DoTC not senescent, but that both senescence and quiescence can be reversed, we can ask which mechanisms are responsible for avoiding regrowth, as well as what specific signals promote quiescence instead of senescence. New research is necessary to address these fundamental questions. An example of an effort to generate new knowledge is provided by focusing on the DREAM protein complex as a mechanism that can act as a switch regulating cell fate [177]. DREAM consists of the retinoblastoma protein (p130 or p107), gene repressor E2F (E2F4 or E2F5), and Multi-vulval class B (MuvB) core of proteins [178]. It functions by suppressing the expression of genes related to cell cycle progression through the induction of senescence or quiescence in a mechanism directly dependent on the initial stimuli. DREAM activity is also regulated by two specific kinases DYRK1A and DYRK1B, highly phosphorylated in tumor cells to promote both cell cycle arrest and high survival [179,180]. Moreover, it has been shown that the specific inhibition of DYRK1 kinases reduces cell viability in cells under dormancy isolated from ovarian spheroids [181]. In the same way that the transforming growth factor-beta (TGF-beta)/bone morphogenic protein (BMP) is involved in cell differentiation, the presence of secreted protein, acidic and rich in cysteine (SPARC), in the bone marrow microenvironment induces DoTC positive for senescence markers, including β-gal staining [143]. Additionally, we must consider the specific genetic background of tumor cells as a major factor influencing the cellular fate. Kovatcheva and cols. [170,182] showed that after CDKi treatment (a well-known senescence inducer), liposarcoma cells can enter in senescence or quiescence, depending on the level of expression of ATRX (Figure 1). In the presence of ATRX, CDKi promotes the formation of senescence-associated heterochromatic foci (SAHF) by recruiting HIRA, HP1, and PML. Additionally, cells that enter in quiescence after CDKi treatment can be senoconverted to senescent cells by downregulating MDM2. Interestingly, the senoconversion process (or the specific conversion from quiescence to senescence) is observed in normal muscle quiescent stem cells and can be promoted by increasing the p16 expression [35] or by genetically and pharmacologically blocking autophagy with bafilomycin A1 [57]. In the cancer context, Buczacki and cols. [183] have shown that senoconversion can be achieved in DoTC cells using small molecules. In this study, putative dormant quiescent CRC cells were induced to senescence through the inhibition of Wnt and Hedgehog pathways by using itraconazole antifungal treatment. This study emphasizes the feasibility of using pharmacological agents to induce senescence in quiescent DoTC and opens the possibility of exploiting synthetic lethality by combining seroconversion agents with other drugs, such as senolytics.

Altogether, these examples highlight the existence of pivotal and additional molecular mechanisms that define the fate of DoTC. To completely understand this process, one must explore deeper to discover the underlying regulatory molecular mechanisms that may be eventually translated into novel therapeutic interventions.

## 10. Microenvironment Influences Dormancy and Senescence

### 10.1. Microenvironment and Dormancy Induction

Many physiological processes are intimately related to the microenvironment, especially relevant due to its role in regulating dormancy control and reawaking tumor cells. Specific tumor cells are prompted to enter dormancy in different niches, but long-term dormancy can be acquired in niches from (a) bone marrow, (b) the lung, (c) perivascular tissue, and (d) the “inside of tumor”. There are specialized spaces within these tissues capable of sheltering stem cells, and it has been proposed that DoTC share the same niches. Additionally, all niches are characterized by an elevated concentration of members of TGF-β/BMP family factors secreted by either cells neighboring the niche or by tumor cells. From these four niches, the most prolonged dormancy seems to be induced by the perivascular and bone marrow niche (reviewed in [72,184]) (see Table 1 for a summary of microenvironment trigger signals associated with dormancy and senescence). These stem cell niches are primarily occupied by mesenchymal stem/stromal cells (MSCs) [185]. MCS exhibits a physiological secretome mainly composed of HGF, TGF-β, CCL2/5, IL-6, VEGF, TSG-6, PGE2, and galectins 1/9 ([186] and reviewed in [187]), an anti-inflammatory environment protective against pro-inflammatory stimuli like INF-γ [188] or in 3D conditions [189]. During dissemination, the prostate DoTC target and occupy the hematopoietic stem cell (HSC) niche in the bone marrow [190]. In the osteoblastic niche, tumor cells require the expression of the Axl receptor to activate it in response to microenvironmental growth-arrest specific 6 (GAS6) to maintain the dormancy state. The Gas6-Axl axis is also necessary for TGF-β2-mediated quiescence. Interestingly, low Axl expression is correlated with longer survival in prostate cancer patients since it is not observed in tumor cells in either primary or in metastatic lesions [121,150,191]. Activation of the axis Gas6-Axl in primary vascular cells delays senescence through PI3K/Akt/FoxO signaling [192,193], although more research is required to understand if this mechanism is relevant in tumor evolution.

### 10.2. Senescent Secretome in a Dormancy Context

As previously mentioned, senescent cells possess the capacity to remodel and reorganize the microenvironment through SASP, and although they share many mutual components with the MCS secretome, they have more prominent pro-inflammatory factors [53,86,194,195]. It is therefore justifiable to believe that there is a relationship between senescence and dormancy at many levels, despite the possibility that senescence could be considered another kind of dormancy. In this way, an interesting report demonstrated that dormancy is induced in triple-negative breast cancer cells through entosis of a coculture with MSC in a 3D format [144]. These results suggest that tumor cells swallow MSC and then acquire a dormant-senescent phenotype, characterized by cell cycle arrest; a high resistance to starvation; and, interestingly, a secretome similar to SASP that includes CSF3 (granulocyte colony-stimulating factor), PTGS2 (COX2), TNF-α, IL1-α, IL1-β, IL-6, IL-8, CXCL1, CXCL2, CXCL10 (IP10), and CCL20, as well as the antiapoptotic factor IFI6 and the tumor suppressor EGR1. Similarly, p53 wild type breast tumor cells induced to senescence with doxorubicin activate phagocytosis and macrophage-like programs promoting a higher survival capacity during long dormancy periods after engulfing proliferative neighbors [196]. Factors present in the microenvironment can modulate senescence induction. In this context, prostate dormant cells isolated in vivo secrete high levels of protein acidic and rich in cysteine (SPARC). SPARC induces the expression of BMP in bone stromal cells and promotes a reversible senescence state characterized by high levels of p38 and p21 (see Table 1). High SPARC promoter methylation negatively correlates with the disease-free survival of prostate cancer patients. On the other hand, Noggin expression in tumor cells or its presence in the microenvironment suppresses BMP7-BMPR2 signaling and supports cancer cells to avoid dormancy [143].

### 10.3. Immune Recognition and Clearance of Dormant and Senescent Cells

The interaction between the human immune system and cancer is complex and highly regulated. The immune system is able to identify specific surface receptors (e.g., major histocompatibility complex (MHC) molecules class I and II) and specifically eliminate cancer cells. Immunotherapy by immune checkpoint blockade targeting tumor-specific neoantigens, by monoclonal antibodies, and by T-cell transfer therapy is now a reality for patients with solid and liquid tumors [197,198,199]. Patients undergoing immunosuppressive treatments have a higher incidence of cancer [199], which suggests that cells prone to tumor development might be in a dormant state; an event observed in bone marrow from patients with breast cancer and accompanied by over-activated immune cells, including natural killer (NK) cells and T lymphocytes [199]. In healthy individuals (and not in melanoma patients), melanoma-specific T lymphocytes displaying a strong reactivity against peptides of melanoma antigens Tyrosinase–MAGEA3–Melan-A/Mart-1–Pmel 17gp100 and NY-ESO-1 have been identified [200]. These data support the existence of endogenous autoimmunity against melanoma, preserving tumor dormancy and protecting from malignant cell growth. T lymphocytes and NK cells are relevant as they trigger cytotoxic responses to regulate the equilibrium between metastatic dormant cells and the immune system. Moreover, dormancy of immune cells promotes cancer cell growth arrest and angiogenic control. Immunotherapeutic interventions against dormancy and senescence cell fates can be considered suitable approaches for targeting primary tumors and metastasis. Coupled to these observations, evidence suggests that immune clues on the microenvironment promote dormancy on tumor cells. Immune cells present in some potential dormant niches directly impact dormancy physiology through the secretion of specific factors (reviewed in [201,202]). For example, CD8+ T cells can induce tumor cell dormancy via the production of IFN-γ, and CD4+ T cells produce CXCL9 and CXCL10, avoiding micro angiogenesis and promoting hypoxic-induced dormancy. Finally, it has been observed that perforin secreted by natural killers induces a long period of dormancy in vitro and in vivo. It is therefore predictable that this kind of microenvironment allows dormancy to be maintained for long periods of time.

On the other hand, senescence limits tumor growth and affects immunosurveillance. Evidence suggests that in early premalignant lesions, an increased number of senescent cells are found, revealing the intrinsic tumor suppression nature of senescence. Through their secretome, oncogene-induced senescent hepatocytes in vivo are capable of activating the immune system for its own clearance and this depends on the intact CD4(+) T-cell-mediated adaptive immune response and the tuned activity of CCR2+ myeloid cells. On the contrary, the impairment of immune senescent surveillance results in the growth of hepatocellular carcinoma tumors in murine models, and it is a poor prognosis marker for patients [203,204].

If senescent cells are well-recognized and cleared by immune cells, there are situations in which the immune system is incapable of recognizing them. For example, in individuals with impaired cell cytotoxicity, the accumulation of senescent cells in tissues is recognized and accompanied by signs of premature aging [204]. Moreover, in aged organisms, dermal senescent fibroblasts expressing high levels of HLA-E can evade immune clearance. These MHC surface molecules interact with inhibitory receptor NKG2A from NK and highly differentiated CD8+ T, inhibiting the immune response against them [205].

In the cancer context, some of the mechanisms that senescent cells use for immune evasion have been identified. One of them (shared with normal senescent cells) includes NKG2D, which, like Matrix metalloproteinases (MMPs) (a factor included in the senescent secretome), disrupts immunosurveillance by paracrine action in the microenvironment [206]. Moreover, the senescent secretome from Pten-null tumors can establish an immunosuppressive microenvironment by the activation of Jak2/Stat3 and downregulation of PTPN11/SHP2 pathways [68].

The senescent secretome is a complex issue, and its composition depends on cellular types and senogenic stimuli [207,208]; however, target therapy, specifically the use of CDK inhibitors, promotes the awaking of cytotoxic T-cell immunity directed by the production of type III interferons induced by the production of double-chain RNA molecules inside the tumor senescent cell [209]. Furthermore, the use of CDKi plus immune activators (e.g., kinase inhibitors, PD-1 inhibitors) increases the survival of treated human xenograft models [210,211,212]. This combination (CDKi abemaciclib with anti PD-1 antibody pembrolizumab) is currently being used in clinical trials to test the efficacy in glioblastoma (NCT04118036) and in HR+ and HER2- metastatic breast cancer (NCT02779751) [212].

### 10.4. The Awaking of Dormant Cells is Primarily Promoted by the Microenvironment

Finally, a fundamental question remains to be addressed: what are the main pathways that trigger the awaking of DoTC? A potential explanation is the action of specific signaling molecules such as Noggin. Another interesting example is the activity of Coco in lung metastases. Coco functions in a similar way to Noggin as a BMP inhibitor that promotes the reawaking of dormant breast tumor cells, leading to a state of high stemness by activating the expression of Nanog, Sox2, and Taz. Conversely, BMP4 completely suppresses the stemness profile expression [119]. Another possibility causative of DoTC reawaking is inflammation, specifically if disbalance [212] between anti-inflammatory and pro-inflammatory factors occurs in the dormant niche. In D2A1 slow-cycling cells, in vivo isolated dormant cells were exposed to Lipopolysaccharides (LPS) endotoxins. The inflammation produced was sufficient to promote proliferation in DoTC and increase the number of recurrences of lung tumors [125]. Similarly, D2.0R dormant cells exposed to tobacco smoke or LPS inflammation stimuli are characterized by the formation of neutrophil extracellular traps (NETs) and subsequent matrix remodeling by MMP9 and NR (Neutrophil elastase). The NET-mediated proteolytic remodeling of laminin revealed an epitope sensed by DoTC through integrin α3β1, leading to proliferation by the activation of FAK/ERK/MLCK/YAP signaling [213]. Finally, a study performed in the Mcf7 cell line induced to dormancy by culturing in the presence of FGF-2 revealed the possible action of pro-inflammatory cytokines such as IL-6, IL-8, and TGF-β1. Furthermore, dormant cells co-cultured with SASP from bone marrow stromal cells (e.g., oxidation, hypoxia, or estrogen deprivation) proliferate after 6 days in vitro [214]. Consistent with this evidence, it has been proposed that the capacity of senescent cells to escape from cell cycle arrest is due to exposition to SASP, especially tumor senescent cells exposed to different chemotherapeutics [6,73,215] which retain the capacity to regrow tumors in vivo [74]. This kind of recurrence occurs after long periods of time. During aging, TGF-β signaling induces senescence in bone-derived mesenchymal stromal/stem cells accompanied by the activation of p21 [59,163], and in long-term recurrences, aging could be the major risk factor of recurrence associated with a chronic inflammation state called inflammation [216].

## 11. Two Models of Tumor Dormancy

Up to this point, considering the existence of more than one kind of dormancy is not an unwarranted idea. Based on the existing evidence, one can divide cancer cell dormancy into two different phenotypes with regards to evolution: (1) Short-term dormancy, putatively caused by quiescent cells and coinciding with clinic evidence of relapse after months or even a few years of disease-free survival, and (2) long-term dormancy, putatively caused by senescent cells and coinciding with clinical evidence of relapse after 10 years or more of disease-free survival. The incidence of one or another sort of dormancy could also depend on two main factors: the origin of the primary tumor and the niche in which the dormant cells engage (Figure 1 depicts the factors involved in short-term and long-term dormancy). Under this classification, a clear example of senescent-induced dormancy is presented by prostate tumor cells, which present a long time period of dormancy, target bone marrow niches, and are positive for SA-β gal staining. In the same manner, colorectal cancer tumor cells could be relevant as a model for quiescent dormant cells due to their rapid regrowth capacity. Evidence from lung, ovarian, and breast tumors suggests that both short-term and long-term phenotypes could be acquired in this kind of cell, depending on the microenvironment and the regulatory pathways within the cells. According to this notion, BMP and TGF-β signaling backed by p53 (or other tumor suppressors) promote preferential senescence; as opposed to NR2F1 and DREAM, which drive quiescence. To test this working hypothesis, we propose focusing efforts on the systematic analysis of clinical observations and patient samples in an unbiased manner. Knowledge of underlying molecular mechanisms regulating cancer cell assignments to types and states would provide opportunities to develop novel therapeutic strategies to target cancer outcomes.

## 12. Senolytic Therapy Is Beneficial in Cancer

Senolytic therapy is defined by the selective clearance of senescent cells in aging and diseased tissues [217]. Senescent cells accumulate during aging in different tissues, promote inflammation, and often elicit deleterious effects [218]. The selective elimination of senescent cells promotes an improvement in physiological performance in aging-related diseases [219,220,221,222]. Cells from transgenic mice overexpressing the tumor suppressor p16INK4a are sensitive to pharmacological elimination. Chemotherapy not only attenuated aging-associated disorders, but also extended the lifespan [219]. In cancer, an increase of senescent cells in normal tissue is normally observed after treatment with chemotherapeutics. Treatment with senolytic compounds targeting cell death regulator caspases (e.g., BCL-2 and BCL-XL) improves the health span, decreases metastasis, and increases the rate of disease-free survival after chemotherapy [220,222]. This data highlights the possibility of using senolytic therapy not just as secondary treatment, but as a “first-line” treatment in combination with senogenic treatment using a two-hit strategy targeting TIS cancer cells: irreversible senescence induction plus the selective elimination of senescent cells ([223,224,225] and reviewed in [226,227,228]).

### 12.1. Experimental Evidence Highlights the Efficacy of Senolytics as a Cancer Treatment

The selective elimination of senescent cells extends the survival of mice bearing TIS tumors, but not of those mice with a tumor resistant to senescence [223]. In this context, specifically targeting senescent human melanoma cells (i.e., SK-MEL-103) in xenografted tumors demonstrated antitumoral efficacy. The combination of senogenic compound Palbociclib with doxorubicin or navitoclax formulated in 6-mer galactooligosaccharides (referred to as GalNP) nanoparticles shows a remarkable advantage over each treatment alone at reducing tumor growth, and secondary effects like cardiotoxicity and thrombocytopenia [229]. By taking advantage of this approach, a synthetic lethality approach can be used to eliminate epithelial ovarian cancer and Triple Negative Breast Cancer (TNBC) cells transiently induced to senescence with poly ADP ribose polymerase (PARP) inhibitors and a panel of senolytics [230].

In studies from our laboratory, cardiac glycosides (CG) acting as sodium-potassium pump blockers were identified as a new broad class of senolytics. Combination treatment with senogenic gemcitabine and CGs of non-small lung carcinoma xenografted tumors show a significant efficacy at decreasing tumor volume in comparison with each agent alone. This two-hit combination therapy was efficient at diminishing the size of patient-derived xenograft (PDX) tumors from triple-negative breast cancer using doxorubicin as a senogenic and digoxin as a senolytic, offering a clear advantage over each compound alone [231]. Moreover, another group in an independent manner also identified GCs as senolytics. As an intrinsic tumor suppressor mechanism, normal cells go to senescence after oncogenic stimuli, but remain in tissues as preneoplastic cells. Treating mice with ouabain reduced the occurrence of cancer by the direct elimination of senescent cells in two different models of genetic carcinogenesis, opening the possibility of also using senolytics as a cancer prophylactic treatment [232].

A recent study from Wang and cols. [80], using kinome-focused genetic screening, identified a DNA-replication kinase CDC7 as a target for senescence induction in p53 null liver cancer. The pharmacological inhibition of CDC7 induces senescence in solid tumor models. Moreover, using chemical screening, they found that the pharmacological inhibition of mTOR promotes senolysis in CDC7-inhibited senescent cells. This combination showed beneficial effects in a xenograft and in immune-competent, somatic mouse models of hepatocellular carcinoma by promoting tumor regression and the highest overall survival.

As these studies indicate, this therapeutic strategy offers an enhanced efficacy during and after treatment, with special relevance for tumors inherently resistant to conventional chemotherapy and with a high rate of metastasis and recurrence. In the shreds of evidence reviewed, we can list another point in favor of this approach. Treatment with senolytics with suitable timing can ensure the elimination of cells, with the possibility of preventing senescence and acquiring the most aggressive stem-like phenotype (autocrine stemness) [23], by avoiding the rise of subpopulations with a high stemness through SASP from tumor cells and non-malignant cells from tumor-surrounded tissues [52,53,54,233]. In the same way, clearing senescent cells existing in tumors can prevent migration and possibly metastasis [86,88]. Quiescent DoTC can be possibly senoconverted to senescent cells, which may make them sensitive to senolytics [183]. However, it will be necessary to develop tools and methodologies to identify new pharmacological senoconverters that can facilitate such differentiation. The low rate of recurrence after chemotherapy observed in these studies suggests that targeting the senescent-dormant phenotype with senolytics might be a front-line strategy to reduce the metastatic recurrence. We hypothesize that a combination of senogenic and senolytic treatments might have a beneficial effect on cancer evolution by preventing metastasis. To test this hypothesis, efforts should be directed to demonstrating the broad efficacy of the selective elimination of senescent-dormant cells on invasion and metastasis progression from dormant solid tumors, for example, by targeting dormancy drivers (Figure 1).

### 12.2. Senolytics in Clinical Development

The first in-human trial of senolytic drugs in patients with idiopathic pulmonary fibrosis (IPF) data is encouraging and indicates the feasibility of larger clinical trials [234]. In this pilot study, investigators enrolled 14 adults diagnosed with stable, primarily mild-to-moderate IPF. Each participant received a combination of senolytic drugs, dasatinib, and quercetin (DQ), U.S. FDA-approved for other indications. The mobility of participants in a six-minute walk distance test was improved by an average of 21.5 m. DQ treatment has shown efficacy at eliminating senescent cells originating from different cell types and improving pathology in animal models of Alzheimer’s disease. Indeed, in a second study performed in nine patients with diabetes-related kidney disease, it has been demonstrated that DQ senolytic treatment not only removed senescent cells from the body, but also alleviated insulin resistance, cell dysfunction, and other processes that cause disease progression and complications [235]. Although these preliminary data are promising, they should be interpreted with caution and further study is required due to limitations in terms of the sample size and lack of a placebo group. The identification of safe senolytics within the approved drugs may facilitate the development of such studies in larger randomized and controlled trials.

## 13. Outlook

The overall scope of this review was to collect relevant evidence on the state-of-the-art of cancer cell fate specification in development and progression. The development and translational investigation of new therapies or treatments for cancer can be expedited by the latest precision medicine and artificial intelligence approaches. This can facilitate drug discovery with a direct input into the regulatory science and industrial technological innovation pipeline. Understanding the continuum of proliferation, senescence, stemness, dormancy, quiescence, and cell cycle re-entry states and contexts of tumor formation and metastasis in relevant 3D models and organ-/lab-on-a-chip might provide a platform to increase the success rate in translating new solutions (e.g., diagnostics, treatment, and follow up) into real clinical innovative approaches that ultimately benefit patients.

## Figures and Tables

**Table 1:** Origins and consequences of cancer cell fate: proliferation, senescence, stemness, dormancy, quiescence, and cell cycle re-entry.

Fate	Genetic Origin	Trigger Signal	Tumor Mass/Tumor Cell Outcome	Current and Prospective * Therapeutic Interventions
Stemness	Stemness: Nanog, Sox2, TGF-β, Wnt, OCT4 EMT: TWIST1, SNAI1	Microenvironment factors Hypoxia Loss of senescence	Tumor growth Survival and drug resistance Invasion and migration Metastasis	Differentiation therapy Targeted therapy *
Quiescence	Cell cycle regulators: p21, p27 Others: TGF-β, HIFα1, Gas6	Microenvironment factors Hypoxia Starvation	Survival and drug resistance Invasion and migration Metastasis	Targeted therapy Geroconversion therapy + senolytics *
Dormancy	Dormancy pathways: NR2F1, SPARC, TGF-β Stress response: p38, mTOR, ATFα6 Proliferation: ↑p21, p27, ↓ERK, Myc, GAS6 Stemness: Wnt, Rank, Nanog, Sox9	Microenvironment clues	Metastasis Tumor re-growth Survival	Awaking therapy Pro dormancy therapy * Targeted therapy Geroconversion therapy + senolytics *
Cell cycle re-entry	From quiescence: Coco, Nogging, Taz, FAK From senescence: ↓p53, Suv39H1, ↑MDM2	Microenvironmental signaling Loss of tumor suppressors	Tumor relapse Cancer stemness	Second-line chemotherapy Immunotherapy Senolytics *
Senescence	Tumor suppression activation: p53, p38, p16, p21, p27, pRb, ATRX, PML, p38 Secretion pathways: BMPs, TGF-β, NFƘB, JAKs IL-6	Genotoxic stress and chemotherapeutics Physical stress Targeted therapy	Tumor relapse Cancer stemness	Senolytics *
Proliferation	Oncogene activation: RAS, ERK, Cyclin D1	Oncogene driver mutation Tumor suppressor gene mutation	Tumor growth	First-line chemotherapy Immunotherapy

* Therapies in pre-clinical stages.

## Abbreviations

AML	Acute myeloid leukemia
BMP	Bone morphogenetic protein
BMPR	Bone morphogenetic protein receptor
CCL	chemokine (C-C motif) ligand
CDK	Cyclin-dependent kinase
CSC	Cancer stem cells
CSF3	Granulocyte colony-stimulating factor
CTC	Circulating tumor cells
CXCL	C-X-C motif chemokine
DDR	DNA damage response
DoTC	Dormant tumor cells
DREAM	Dimerization partner, RB-like, E2F, and multi-vulval class B
DTC	Disseminated tumor cells
DYRK	Dual specificity tyrosine-phosphorylation-regulated kinase
EGF	Epidermal growth factor
EMT	Epithelial-mesenchymal transition
ERK	Extracellular signal–regulated kinase
FAK	Focal adhesion kinase
Gas6	Growth-arrest specific 6
GDF	Growth/differentiation factor
HGF	Hepatocyte growth factor
IGF	Insulin-like growth factor 1
IGFR	Insulin-like growth factor receptor
iPS	Induced pluripotent stem cell
MSC	Mesenchymal stem/stromal cells
MuvB	Multi-vulval class B
MYLK	Myosin light-chain kinase
NR2F1	Nuclear Receptor subfamily 2, group F, member 1
OIS	Oncogene-Induced Senescence
RANK	Receptor activator of nuclear factor κ B
RCCC	Rapid-cycling cancer cells
SAHF	Senescence-associated heterochromatin foci
SASP	Senescence associated secretory phenotype
SCCC	Slow-cycling cancer cells
SPARC	Secreted protein acidic and rich in cysteine
TGF-β	Transforming growth factor beta
TGS	Tumor necrosis factor-inducible gene
TIS	Therapy induced senescence
TNF-α	Tumor necrosis factor alpha
VEGF	Vascular endothelial growth factor
YAP	Yes-associated protein
